# From gene expression to causal associations: investigating the role of ferroptosis in cataract development

**DOI:** 10.1186/s12920-025-02177-6

**Published:** 2025-07-01

**Authors:** Chen Li, Xian-bing Yuan, Yi-cheng Lu, Zi-yue Song

**Affiliations:** 1 Department of Ophthalmology, the First Affiliated Hospital of Soochow University, Shizi Street 188, SuZhou, Jiangsu Province 215006 China; 2 School of Clinical Medicine, Medical College of Soochow University, SuZhou, Jiangsu Province 215006 China

**Keywords:** Cataract, Ferroptosis, Hub genes, *TIGAR*, *IL6*, Mendelian randomization

## Abstract

**Background:**

Cataracts are one of the most prevalent blinding eye diseases globally, and ferroptosis may be involved in its pathogenesis; however, the precise mechanisms remain unclear. We therefore aimed to identify ferroptosis-related genes (FRGs) related to cataracts and assess their causal association.

**Methods:**

We downloaded two gene expression profile datasets of patients with cataracts and gathered the FRGs from the MSigDB and GeneCards databases. This allowed us to find the ferroptosis-related differentially expressed genes (FRDEGs). The potential functions of these FRDEGs were explored using Kyoto Encyclopedia of Genes and Genomes (KEGG), gene ontology (GO), and gene set enrichment analysis (GSEA). A protein–protein interaction (PPI) network was established, and hub genes were screened. Additionally, potential diagnostic markers were identified by RT-PCR validation. Finally, a Mendelian randomization (MR) study was performed to ascertain the causal impact of FRDEGs on cataracts.

**Results:**

Nineteen FRDEGs were identified by overlapping DEGs with FRGs. GO, KEGG and GSEA showed that the FRDEGs were associated with oxidative stress, *IL17* signaling pathway, and glutathione metabolism. Nine hub genes were identified using the PPI network and five algorithms in Cytoscape. The RT-PCR results validated *TIGAR*,* IL6*,* ATF3*, and *TNFAIP3* as potential biomarkers.

**Conclusion:**

*TIGAR* and *IL6* were identified to be causally associated with cataracts. Inverse variance weighting revealed that *TIGAR* decreased the risk of cataracts, whereas *IL6* increased the risk of cataract. Our research identified ferroptosis-related hub genes in cataracts, providing valuable insights for pre-symptomatic diagnosis and contributing to our understanding of the molecular mechanisms of cataract risk genes.

**Supplementary Information:**

The online version contains supplementary material available at 10.1186/s12920-025-02177-6.

## Background

Cataracts are common ocular diseases mainly characterized by clouding of the lens, leading to vision loss or even blindness. Globally, cataracts are a major contributor to visual impairment, particularly in the older adult population, where their incidence is high [1, 2]. Despite extensive research, the exact cause of cataracts remains elusive. Increasing evidence indicates that the accumulation of reactive oxygen species (ROS) and an imbalance in lens redox homeostasis are factors initiating the development of cataracts. In the equatorial region, lens epithelial cells (LECs) are the initial site of oxidative stress attack, and under these conditions, they eventually differentiate into fibroblasts; oxidative stress reduces their vitality, resulting in lens opacification [3–5]. Interestingly, despite severe disruption of redox homeostasis, there was no evident cell death in the lenses of patients with cataracts. This raises questions about alternative mechanisms of cell death.

Ferroptosis, a cell death mechanism distinct from apoptosis or necrosis, is characterized by iron-dependent regulatory cell death and the accumulation of intracellular lipid ROS [6, 7]. Morphologically, it is characterized by mitochondrial shrinkage and the disappearance of mitochondrial ridges, as opposed to chromatin aggregation and the formation of apoptotic bodies [8]. Ferroptosis is currently widely studied in ocular disorders such as glaucoma, age-related macular degeneration, diabetic retinopathy, high myopia, and Graves’ orbitopathy [9–13]. Notably, there is a growing interest in understanding the role of ferroptosis in cataract development. Some studies have indicated that the lenses of patients with cataracts show increased ROS formation, increased lipid peroxidation, and the accumulation of intracellular redox-active iron, which are the three markers associated with ferroptosis during the process of cataract formation. Wei et al. demonstrated that ferroptosis could be induced in LECs through treatment with an inducer, and that the concentration of glutathione strongly affected their sensitivity. Their study also revealed that LECs had high sensitivity to ferroptosis, with a positive correlation to the extent of cell aging. Transcriptome analysis corroborated the upregulation of genes related to iron entry and the downregulation of genes associated with iron exit in aged cells [14]. Therefore, ferroptosis may play a role in the occurrence of cataracts, although the precise mechanism remains unclear. To delve deeper into the molecular mechanism of ferroptosis in cataractogenesis, it is imperative to identify ferroptosis-related biomarkers in cataracts using bioinformatic techniques. Identifying the expression of specific genes in diseases is vital for understanding the microscopic mechanisms underlying cataract development and for determining biomarkers relevant to diagnosis and therapeutic evaluation. Therefore, this study aimed to identify new biomarkers and potential mechanisms associated with ferroptosis in cataracts.

Mendelian randomization (MR) is a trusted technique that has recently gained popularity for deducing underlying causal associations [15]. This technique utilizes single nucleotide polymorphisms (SNPs) as instrumental variables (IVs) to evaluate the causal relationship between exposures and outcomes and has been extensively employed in the study of eye diseases [16–18].

We downloaded two datasets containing the gene expression profiles of individuals with cataracts. Genes associated with ferroptosis were collected from the GeneCards and MSigDB databases. This allowed us to identify ferroptosis-related differentially expressed genes (FRDEGs). We conducted gene ontology (GO), Kyoto Encyclopedia of Genes and Genomes (KEGG), and gene set enrichment analyses (GSEA) of the FRDEGs. To further understand the interactions between these FRDEGs, we constructed a protein–protein interaction (PPI) network. To identify hub genes, we refined our gene screening process using different algorithms in the Cytoscape plugin cytoHubba. Using RT-PCR, we identified potential diagnostic markers that could assist cataract diagnosis and enhance our understanding of the potential role of ferroptosis in cataract development. Finally, through MR analysis, we used genetic variants that were strongly linked to exposure factors as IVs to deduce a causal relationship between ferroptosis-related genes (FRGs) and cataracts.

## Methods

### Data source

Gene expression profile datasets of individuals with cataracts were obtained from the Gene Expression Omnibus (GEO; http://www.ncbi.nlm.nih.gov/geo/) database using the GEOquery package (version 2.66) in R. The datasets GSE153022 [19] and GSE161701 [20] both originated from Homo sapiens and are of the mRNA data types. The dataset GSE153022, with the data platform GPL20795, included 6 samples from human LECs (HLECs), of which 3 were cataract disease samples and three were normal samples. The dataset GSE161701, with the data platform GPL24676, included 12 samples from HLECs, of which 6 were cataract disease samples and 6 were normal samples (Table 1). Datasets GSE153022 and GSE161701 were combined into a unified dataset that included 18 samples, of which 9 were cataract disease samples and 9 were normal samples. All the above data were included in this analysis. To mitigate batch effects, we implemented comprehensive data processing and quality control measures by standardizing data formats and removing outliers while carefully documenting sample grouping and batch information (including measurement batches and experimental dates). Exploratory data analysis using principal component analysis (PCA) was performed to identify and visualize batch effects, followed by batch effect adjustment through linear models, the ComBat algorithm, or quantile normalization methods. Specifically, we employed the ComBat function from the sva package (version 3.46) in R to correct for batch effects. Post-adjustment data were re-analyzed to verify successful batch effect removal through visualization and statistical evaluation. Subsequent analyses were only conducted after confirming appropriate batch effect correction to ensure the accuracy and reliability of results.

**Table 1:** Summary characteristics of GSE153022 and GSE161701 datasets

Accession IDs	Platform names	Number of samples	Sample groups	Publication dates on GEO
GSE153022	GPL20795 HiSeq X Ten (Homo sapiens)	6	3 were cataract disease samples and 3 were normal samples	Mar 23, 2022
GSE161701	GPL24676 Illumina NovaSeq 6000 (Homo sapiens)	12	6 were cataract disease samples and 6 were normal samples	Jan 19, 2022

We gathered ferroptosis-associated genes from two databases: GeneCards (https://www.genecards.org/) [21] and MSigDB (https://www.genecards.org/) [22]. The GeneCards database is a comprehensive resource for human gene information. By using “ferroptosis” as our search term, we identified a total of 549 FRGs. In contrast, the MSigDB database is specifically designed to collect and categorize pre-annotated functional gene sets for GSEA. Using the same search term, “ferroptosis,” we identified 64 additional FRGs. In addition, 60 FRGs were obtained from the literature [23]. Finally, we combined FRGs from the three sources to obtain 562 FRGs. Specific gene names are listed in Table S1.

### DEGs associated with ferroptosis

To identify the underlying role, mechanism, and associated biological characteristics of DEGs in cataract, we initially used the limma package (version 3.54) in R language [24] to extract two datasets comprising cataract (cataract group) and normal samples (normal group) from datasets GSE153022 and GSE161701. Subsequently, these datasets were combined and subjected to PCA before and after merging. PCA is a dimension reduction technique that eliminates highly correlated information from data with higher variance. We then used the limma package to conduct differential analysis on the expression spectrum data of the merged dataset. Genes meeting the criteria of |logFC| > 0.25 and p (adjust) < 0.05 were identified as DEGs. Genes with logFC > 0.25 and p (adjust) < 0.05, were identified as upregulated DEGs, while genes with logFC < -0.25 and p (adjust) < 0.05, were classified as downregulated DEGs. The FRDEGs were identified by intersecting the upregulated and downregulated DEGs from the differential analysis of the combined dataset with FRGs, depicted using a Venn diagram. The results of the differential analysis were visualized using a volcano plot using the R package ggplot2 (version 3.4).

### Functional and pathway enrichment analysis of FRDEGs

GO [25] is a widely used approach for conducting extensive studies on functional enrichment, which includes studying biological processes (BP), molecular functions (MF), and cellular components (CC). KEGG analysis [26] provides a comprehensive database containing valuable information about diseases, genomes, biological pathways, and drugs. In our study, we used the clusterProfiler package (version 4.6) [27] to perform GO and KEGG analyses of the DEGs obtained from FRDEGs. To ensure statistical significance, entry screening criteria were set at *p* < 0.05 and a False Discovery Rate (FDR) value (q) < 0.25. The Benjamini-Hochberg (BH) procedure was employed for *p*-value correction.

### GSEA

GSEA was employed to evaluate the distribution of predefined gene sets in gene lists sorted by phenotype correlation to determine the contribution of different genes to the phenotype [28]. Initially, we arranged the DEGs in the merged dataset based on logFC. Enrichment analysis of all the DEGs was conducted using the clusterProfiler package. The GSEA parameters were set as follows: a seed of 2022, 1000 computations, a minimum of 10 genes per gene set, a maximum of 500 genes per gene set, and the BH method for *p*-value correction. The gene set used in this study was “c2.cp.all.v2022.1. Hs.symbols.gmt [All Canonical Pathways],” obtained from the MSigDB database. For significant enrichment, the criteria were set as p (adjust) < 0.05 and FDR value (q) < 0.25.

### PPI network

The STRING database was used to identify known and predicted PPIs. We employed the STRING database [29], setting the biological species as humans and using a minimum interaction score of 0.400 as the criterion. A PPI network was constructed using the 19 FRDEGs obtained and visualized using Cytoscape (version 3.9.1) [30]. We employed the Maximal Clique Centrality (MCC), Maximum Neighborhood Component (MNC), Degree Correlation (degree), Edge Percolated Component (EPC), and Closeness Centrality (closeness) algorithms in the cytoHubba plugin [31] to calculate scores. Hub genes were identified by intersecting the results of these five algorithms.

### RT-PCR validation of the hub genes

To validate the predictive analysis results, we collected anterior capsule tissues from patients undergoing cataract phacoemulsification surgery for RT-PCR analysis. Patients were divided into two groups: the cataract group consisted of patients with significantly cloudy lenses, and the control group consisted of patients with relatively transparent lenses undergoing lens removal surgery to correct presbyopia. Differences in the expression of hub genes in the anterior capsule tissues of these two patient groups were compared using RT-PCR. The study protocol was approved by the Ethics Committee and Institutional Review Board of the First Affiliated Hospital of Soochow University. TRIzol reagent (Invitrogen) was used to extract total RNA according to the manufacturer’s instructions. The quality of the RNA (RNA integrity number, RIN) was assessed using an Agilent 2100 Bioanalyzer (Agilent Technologies, CA, USA), with a RIN of 8 or higher being deemed suitable for further experiments. Total RNA samples were then reverse-transcribed to cDNA, and RT-PCR was performed using SuperScript III RT (Invitrogen). β-Actin was used as the internal normalization standard, and the relative mRNA expression was determined using the ΔΔCT method. Primers used are listed in Supplementary Table S2. The study procedures have passed the approval of the ethics committee of the First Affiliated Hospital of Soochow University.

### MR

Two-sample MR was employed to investigate the causal link between hub genes and cataract risk using SNPs as IVs. Hub gene data were derived from an accessible genome-wide association study (GWAS) database. Genes that showed positive RT-PCR results were selected for MR analysis. Information on *TIGAR*,* IL6*, and cataracts can be found in the provided links (data on *TIGAR* are available at https://gwas.mrcieu.ac.uk/datasets/prot-a-2979/; data on *IL6* are available at https://gwas.mrcieu.ac.uk/datasets/ebi-a-GCST90012025/; and data on cataracts are available at https://gwas.mrcieu.ac.uk/datasets/ukb-e-366_EAS/). The MR analysis was conducted using the “TwoSampleMR” Package (version 0.5.8), with inverse variance weighting (IVW) applied to evaluate the correlation between hub genes and cataract risk. The MR-Egger test was used for additional sensitivity analysis [15, 32, 33].

### Statistical analysis

All data were analyzed and processed using the R software (version 4.2.1). To assess the statistical significance of continuous variables that followed a normal distribution in the two groups, we employed the independent Student’s t-test. Conversely, non-normally distributed variables were analyzed for differences using the Mann–Whitney U test, also known as the Wilcoxon rank-sum test. For comparison and analysis of categorical variables between the two groups, the chi-square test, or Fisher’s exact test, was used. All *p*-values obtained from these statistical tests were two-sided, and *p* < 0.05 was considered statistically significant.

## Results

**Fig. 1:**
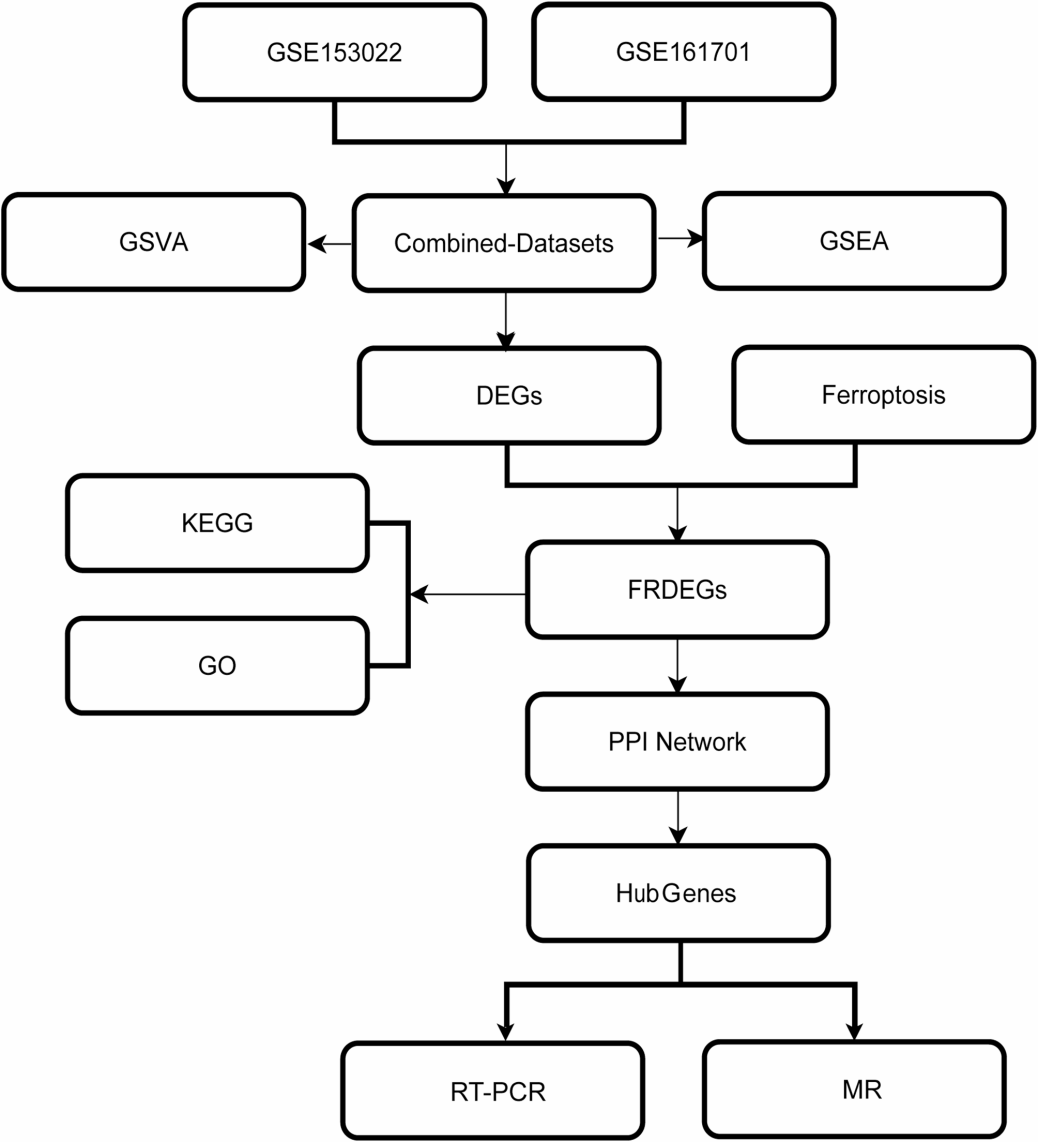
Technology roadmap for this study. DEGs, differentially expressed genes. FRGs, ferroptosis-related genes. FRDEGs, ferroptosis-related differentially expressed genes. GO, Gene Ontology. PPI, protein-protein interaction. KEGG, Kyoto Encyclopedia of Genes and Genomes. GSEA, gene set enrichment analysis. GSVA, gene set variation analysis. MR, Mendelian randomization

**Fig. 2:**
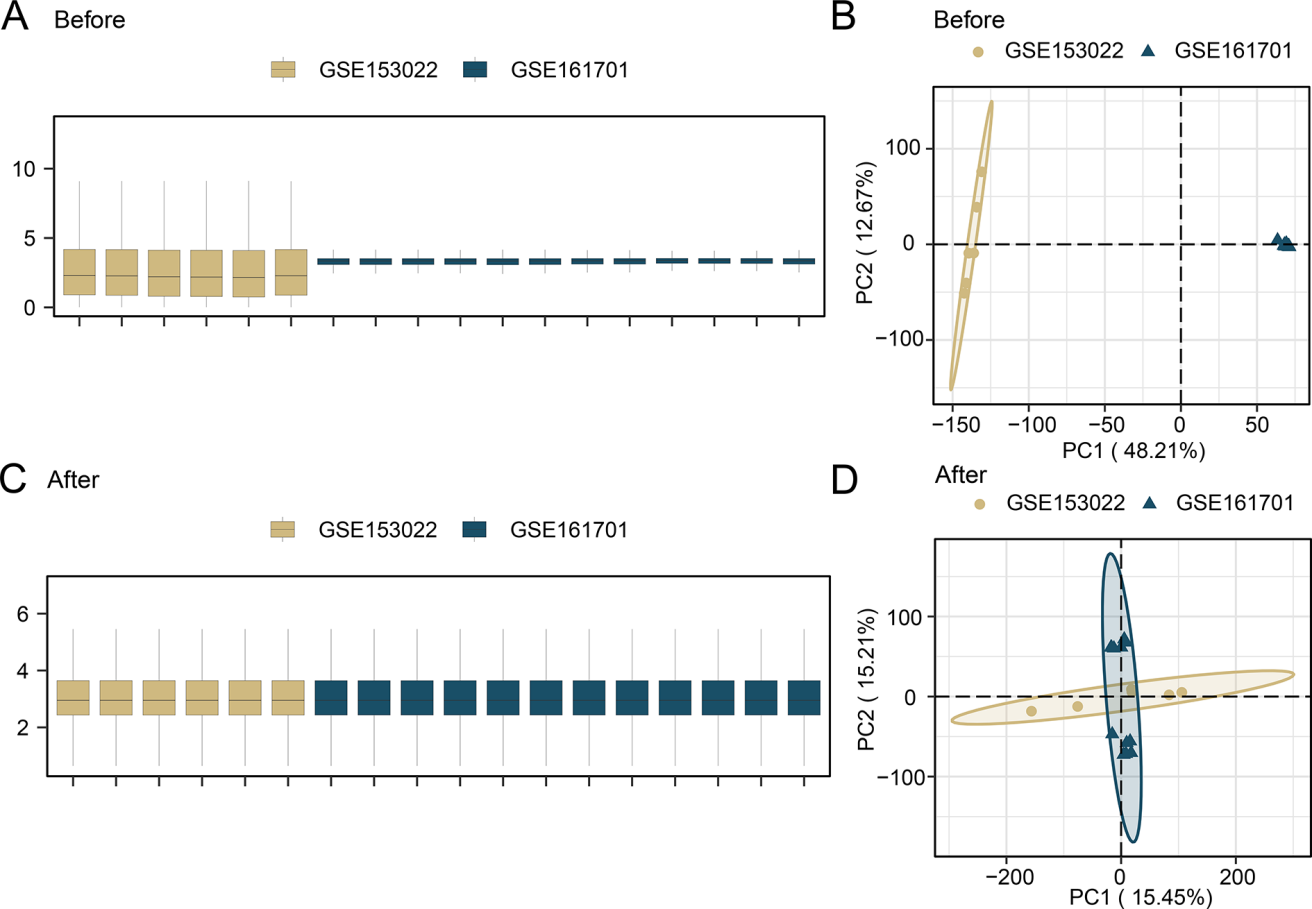
Boxplot and PCA diagrams of merged datasets before and after correction. (**A**) Boxplot of the merged dataset before batch correction. (**B**) PCA diagram of the merged dataset before batch correction. (**C**) Boxplot of the merged dataset after batch correction. (**D**) PCA diagram of the merged dataset after batch correction. PCA, principal component analysis

A flowchart of the study is shown in Fig. 1. First, the data sets were merged and corrected. The R package was employed to eliminate batch effects from the merged cataract dataset, resulting in a consolidated GEO dataset. The datasets before and after batch effect removal were then compared using box plot distribution and PCA graphs (Fig. 2A-D). The box plot distribution and PCA graphs indicate that the batch effects in the samples of the combined dataset were essentially eradicated after batch effect removal.

### Differentially expressed genes associated with ferroptosis

**Fig. 3:**
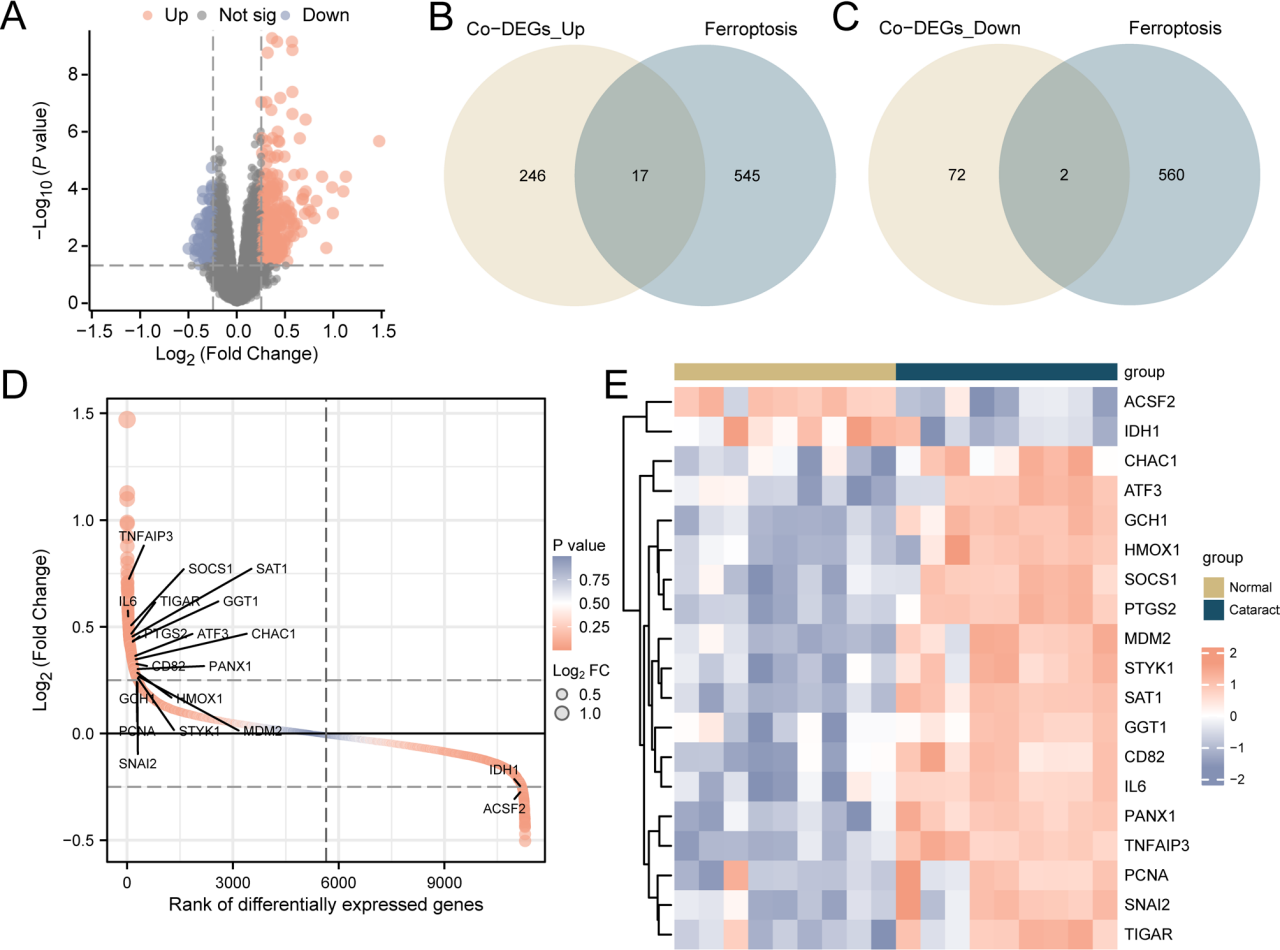
Merged dataset volcano plot of differential sorting, heatmap, and Venn diagram. (**A**) Volcano plot of differential genes in the cataract group and normal group within the merged dataset. (**B**) Venn diagram of upregulated DEGs and FRGs in the merged dataset. (**C**) Venn diagram of downregulated DEGs and FRGs in the merged dataset. (**D**) Differential sorting plot of the cataract group and normal group within the merged dataset. (**E**) Heatmap of FRDEGs in the cataract group and normal group. FRDEGs, ferroptosis -related differentially expressed genes. DEGs, differentially expressed genes. FRGs, ferroptosis -related genes. Co-DEGs, combined datasets- differentially expressed genes

The merged dataset was analyzed to compare gene expression values between the cataract and normal groups. This involved dividing the data into two groups: the cataract and normal groups. A total of 337 genes in the merged dataset met the DEGs threshold of |logFC| > 0.25 and p (adjust) < 0.05. Within this threshold, 263 genes were upregulated, and 74 were downregulated. Differential analysis results were visualized using a volcano plot (Fig. 3A) and a differential sorting plot (Fig. 3D). To identify FRDEGs, FRGs were sourced from the GeneCards and MSigDB databases, as well as existing literature. Phenotype-related genes from these sources were combined to identify the common FRGs. The intersection of all |logFC| > 0.25 and p (adjust) < 0.05 DEGs and FRGs was determined, and a total of 19 FRDEGs (*SAT1*,* TNFAIP3*,* PTGS2*,* GCH1*,* PANX1*,* MDM2*,* CD82*,* STYK1*,* SOCS1*,* IL6*,* HMOX1*,* TIGAR*,* SNAI2*,* CHAC1*,* ATF3*,* PCNA*,* GGT1*,* ACSF2*, and *IDH1*) were obtained, and a Venn diagram (Fig. 3B-C) and a heatmap (Fig. 3E) were drawn.

### Enrichment analysis of FRDEGs

**Fig. 4:**
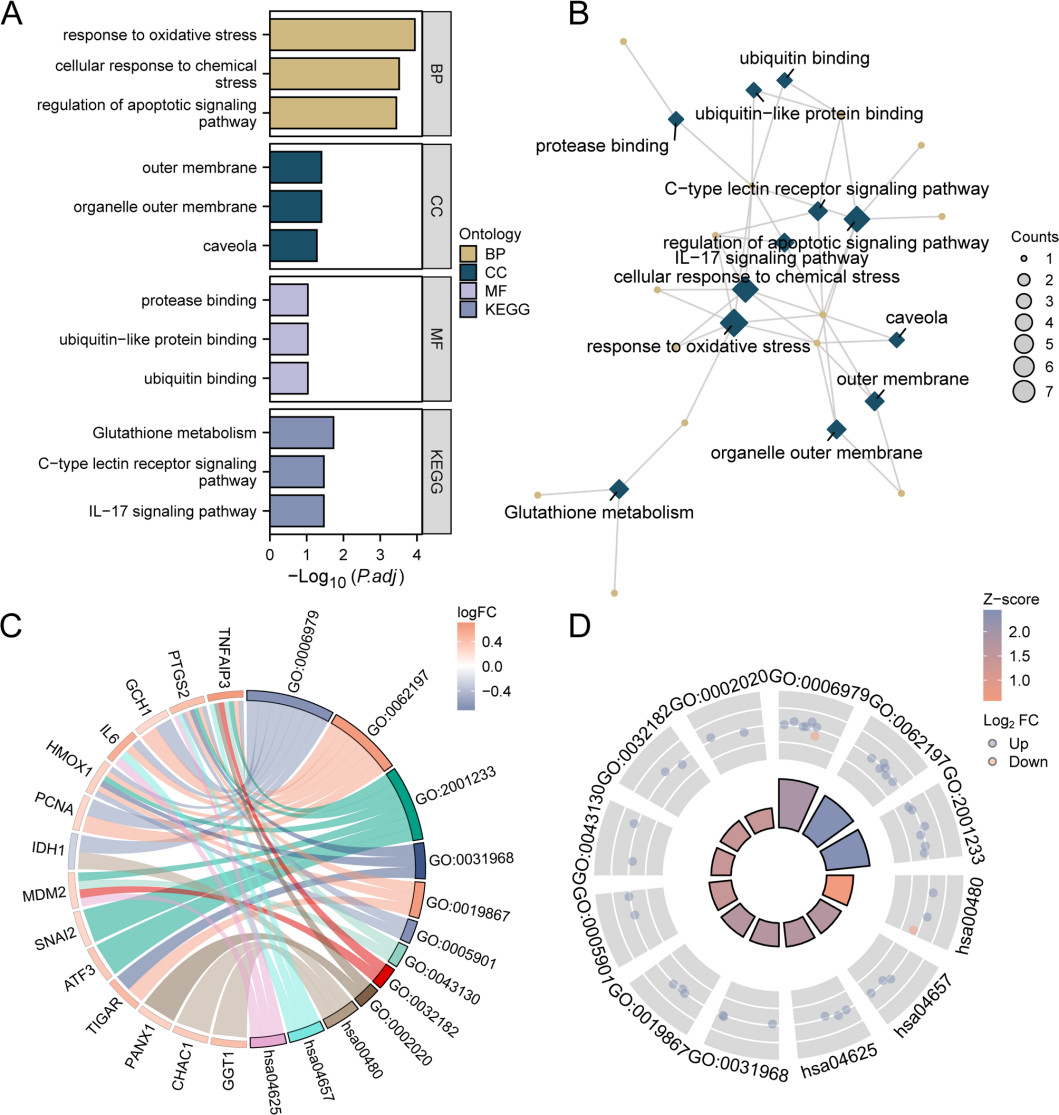
Functional enrichment analysis and pathway enrichment analysis of FRDEGs. (**A**) Bar chart of the results of GO analysis and KEGG analysis of FRDEGs. (**B**) Network graph of the results of GO analysis and KEGG analysis of FRDEGs. (**C**) Chord diagram of the combined logFC results of GO analysis and KEGG analysis of FRDEGs. (**D**) Circle diagram of the combined logFC results of GO analysis and KEGG analysis of FRDEGs. FRDEGs, ferroptosis-related differentially expressed genes. GO, Gene Ontology. BP, biological process. CC, cellular component. MF, molecular function. KEGG, Kyoto Encyclopedia of Genes and Genomes

To analyze the BP, MF, and CC of the 19 FRDEGs, we initially performed GO and KEGG enrichment analyses. The results of these analyses are presented in a bar chart (Fig. 4A). The relationship between the 19 FRDEGs and GO and KEGG enrichment analyses using a network graph is presented in Fig. 4B. The lines in the graph represent the annotations of relevant molecules and entries, where larger nodes indicate entries containing more molecules. Subsequently, GO and KEGG enrichment analyses were conducted with joint logFC on the FRDEGs. This was based on enrichment analysis, involving the logFC values of the FRDEGs in the differential analysis results of the combined dataset and computing the Z-score for each gene. The results of the joint logFC GO and KEGG enrichment analyses are depicted in chord (Fig. 4C) and circle diagrams (Fig. 4D). As shown in Fig. 4A-D, FRDEGs were mainly enriched in BP, such as response to oxidative stress (GO:0006979), cellular response to chemical stress (GO:0062197), and regulation of the apoptotic signaling pathway (GO:2001233); CC, such as organelle outer membrane (GO:0031968), outer membrane (GO:0019867), and caveola (GO:0005901); and MF, such as ubiquitin binding (GO:0043130), ubiquitin-like protein binding (GO:0032182), and protease binding (GO:0002020); and KEGG pathways, such as IL-17 signaling pathway (hsa04657), glutathione metabolism (hsa00480), and c-type lectin receptor signaling pathway (hsa04625). Supplementary Table S3 presents the results of the enrichment analysis.

### Results of the GSEA

**Fig. 5:**
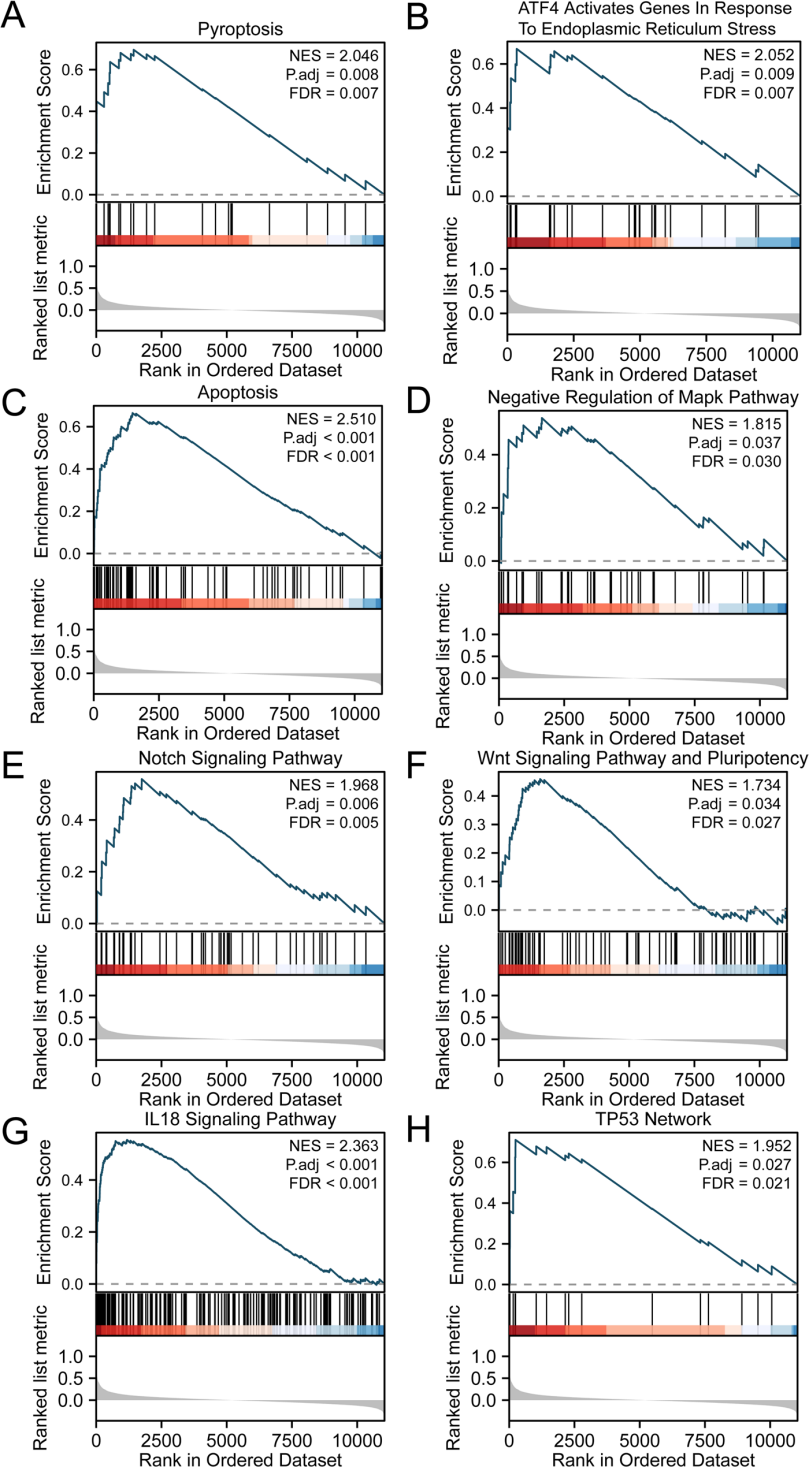
Visualization of GSEA analysis results. A-H. Biological function presentation of GSEA in the merged dataset. GSEA shows genes significantly enriched in REACTOME PYROPTOSIS (**A**), REACTOME ATF4 ACTIVATES GENES IN RESPONSE TO ENDOPLASMIC RETICULUM STRESS (**B**), WP APOPTOSIS (**C**), REACTOME NEGATIVE REGULATION OF MAPK PATHWAY (**D**), WP NOTCH SIGNALING PATHWAY (**E**), WP WNT SIGNALING PATHWAY AND PLURIPOTENCY (**F**), WP IL18 SIGNALING PATHWAY (**G**), WP TP53 NETWORK (**H**). GSEA, gene set enrichment analysis. NES, normalized enrichment scores. FDR, false discovery rate

GSEA was performed to evaluate the influence of gene expression levels on the incidence of cataract. This analysis examined the correlation between the expression of all the genes in the combined dataset and the implicated BP, impacted CC, and applied MF. The findings reveal significant enrichment of all genes in the combined dataset in various pathways such as pyroptosis (Fig. 5A), *ATF4* activation of genes due to endoplasmic reticulum stress (Fig. 5B), apoptosis (Fig. 5C), negative regulation of the MAPK pathway (Fig. 5D), Notch signaling pathway (Fig. 5E), Wnt signaling pathway and pluripotency (Fig. 5F), *IL18* signaling pathway (Fig. 5G), and TP53 network (Fig. 5H) among others. The results are shown in Supplementary Table S4.

### Construction of the PPI network

**Fig. 6:**
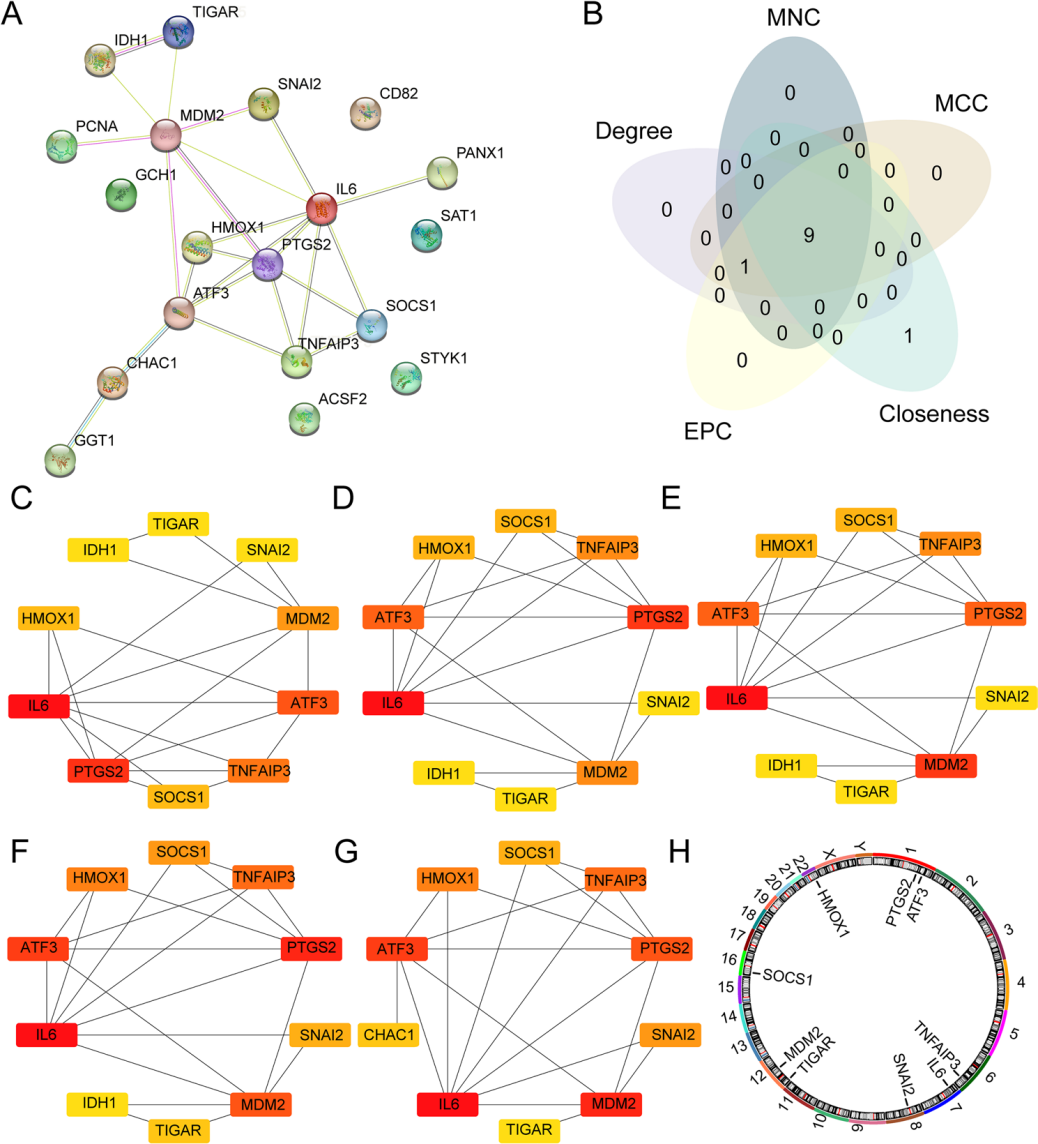
PPI network. (**A**) PPI network of FRDEGs. (**B**) Venn diagram of genes obtained by five algorithms. (**C**) PPI network drawn by top 10 genes in MCC algorithm. (**D**) PPI network drawn by top 10 genes in MNC algorithm. (**E**) PPI network drawn by top 10 genes in Degree algorithm. (**F**) PPI network drawn by top 10 genes in EPC algorithm. (**G**) PPI network drawn by top 10 genes in Closeness algorithm. (**H**) Chromosome location map of hub genes. FRDEGs, ferroptosis-related differentially expressed genes; PPI network, protein-protein interaction network; MCC, maximal clique centrality (**C**); MNC, maximum neighborhood component (**D**); Degree, degree correlation (**E**); EPC, edge percolated component (**F**); Closeness, closeness centrality (**G**); The color of the squares changes from red to yellow, representing scores from high to low

Given the ubiquitous connections between genes and the fact that genes regulating the same BP are closely related, STRING was used to construct a PPI network for a more in-depth exploration of relationships between ferroptosis regulatory genes. The network was visualized using the Cytoscape software. We set the minimum required interaction score at medium confidence (0.400) for PPI network construction using the STRING database (Fig. 6A). Using the MCC (Fig. 6C), MNC (Fig. 6D), degree (Fig. 6E), EPC (Fig. 6F), and closeness (Fig. 6G) algorithms in the cytoHubba plugin to calculate the scores of the sorted genes, the intersection of the five algorithms identified nine hub genes (Fig. 6B). These genes were *IL6*,* PTGS2*,* ATF3*,* TNFAIP3*,* MDM2*,* SOCS1*,* HMOX1*,* TIGAR*, and *SNAI2*. The chromosomal locations were identified (Fig. 6H). The *PTGS2* and *ATF3* genes are located on chromosome 1, the *TNFAIP3* gene is located on chromosome 6, the *IL6* gene is on chromosome 7, the *SNAI2* gene is on chromosome 8, *TIGAR*, and *MDM2* on chromosome 12, the *SOCS1* gene is on chromosome 16, and the *HMOX1* gene is on chromosome 22.

### RT-PCR analysis of hub genes

**Fig. 7:**
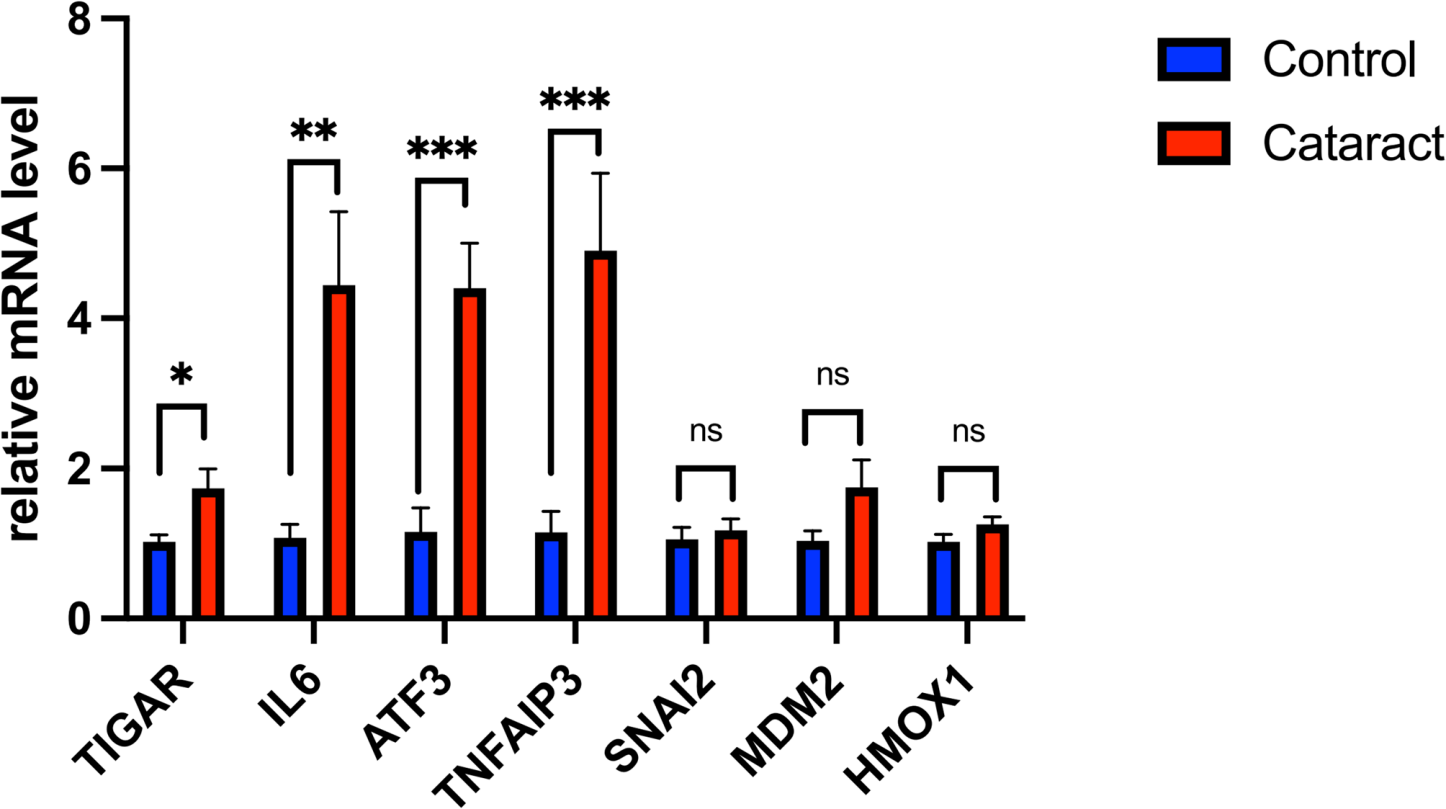
RT-PCR validation of the hub gene between cataract and normal controls. All experiments were carried out three times, and the data were expressed as mean ± SEM (*p < 0.05, **p < 0.01, ***p < 0.001, ns, no significance)

We subsequently performed RT-PCR to measure the relative expression levels of hub genes in both the cataract and control groups. An increased mRNA expression levels of *TIGAR*,* IL6*,* ATF3*, and *TNFAIP3* in the cataract group compared to those in the control group (*p* < 0.05, *p* < 0.01, *p* < 0.001, and *p* < 0.001, respectively) was observed. These genes could potentially serve as diagnostic and prognostic biomarkers. However, there were no notable differences in the expression levels of *SNAI2*,* MDM2*, or *HMOX1* between the two groups (Fig. 7).

### *TIGAR* and *IL6* are associated with the risk of cataracts

**Fig. 8:**
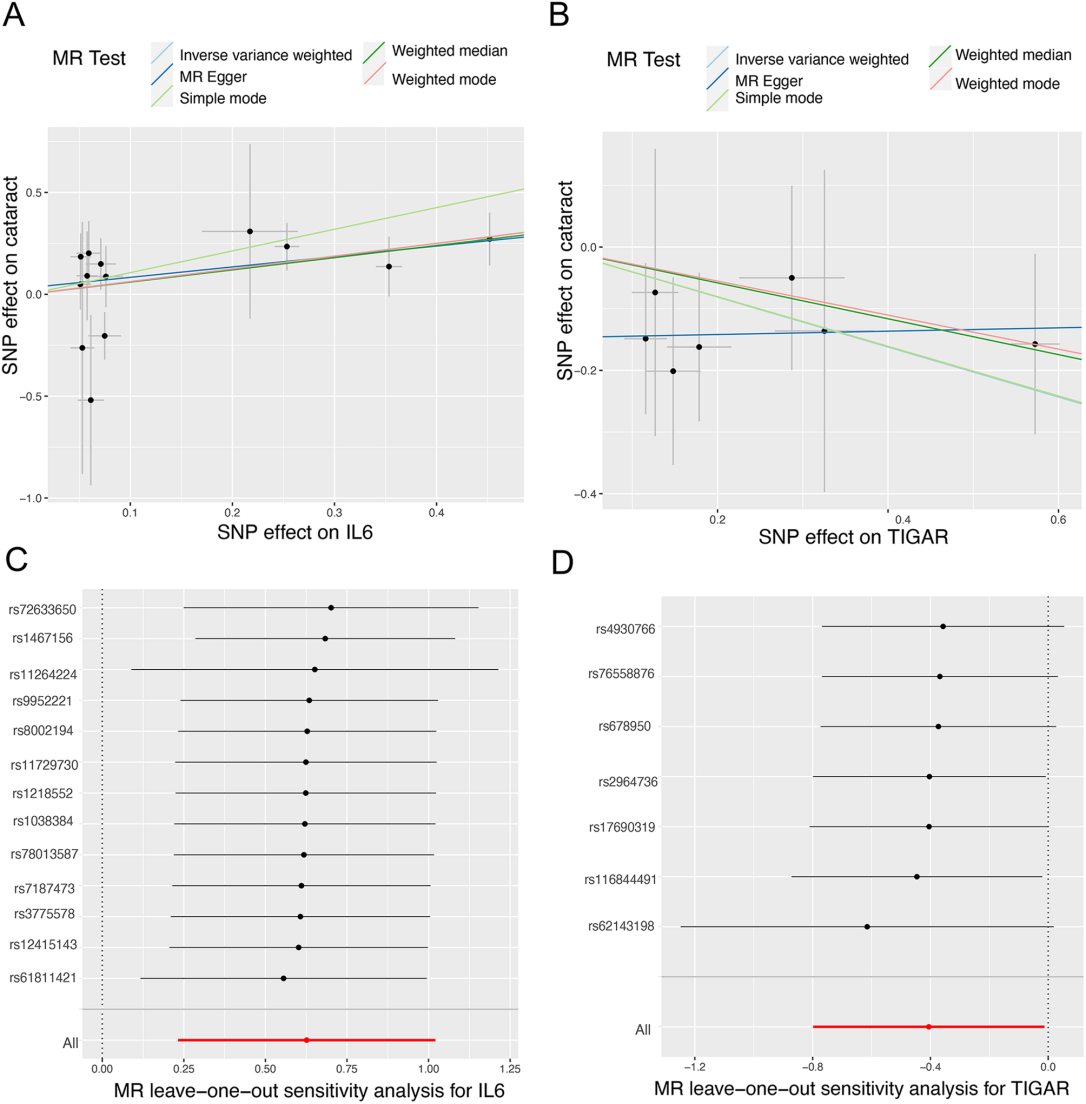
Mendelian randomization study results. (**A**) Scatter plot showing the causal effect of *IL6* on the risk of cataract. (**B**) Scatter plot showing the causal effect of *TIGAR* on the risk of cataract. Each point in the scatter plot represents an SNP. The effect of the same SNP on exposure is placed on the horizontal axis, and the effect on outcome is placed on the vertical axis. The vertical and horizontal lines show the 95% confidence interval (CI) for each SNP. At this point, the slope of the solid line in the plot is each MR estimate. The light blue, light green, dark blue, green, and pink lines correspond to the Inverse variance weighted, Simple mode, MR Egger, Weighted median, and Weighted model methods, respectively. SNP, single nucleotide polymorphism. (**C**) Leave-one-out plot to visualize causal effect of *IL6* on cataract risk when leaving one SNP out. (**D**) Leave-one-out plot to visualize causal effect of *TIGAR* on cataract risk when leaving one SNP out. The leave-one-out plot present how the causal estimates (point with horizontal circle) for the effect of *IL6* and *TIGAR* on cataract were influenced by the removal of a single variant. The bars indicate the confidence interval of MR estimates. SNP, single nucleotide polymorphism

Characteristics of the SNPs in *TIGAR*,* IL6*, and cataracts are presented in Supplementary Table S5. None of the SNPs exhibited weak IVs. Figures 8A and B illustrate the causal effects of each type of genetic variation on cataracts. We evaluated the causal link between *TIGAR* and *IL6* levels and cataracts. Using the primary IVW method, we discovered that *TIGAR* was significantly associated with cataract risk, with an odds ratio (OR) of 0.667 (95% CI = 0.450–0.988, *p* = 0.043). Similarly, the association between *IL6* and the risk of cataracts was significant, with an OR of 1.871 (95% CI = 1.261–2.776, *p* = 0.002). The intercept of the MR-Egger regression did not detect horizontal pleiotropy (*TIGAR*: *p* = 0.225; *IL6*: *p* = 0.615), further suggesting that pleiotropy does not influence the causal effect (Table S6). We re-conducted the MR analysis of the remaining SNPs after eliminating each SNP, as depicted in Fig. 8C and D. These findings were consistent, suggesting that all SNPs had noteworthy causality results. This further implies that *TIGAR*, *IL6*, or cataracts do not have any dominant SNPs, validating the above results.

## Discussion

Cataract, a frequent and blinding ocular disease, severely affects the quality of life. Although cataracts can be treated surgically, surgery carries certain risks and may lead to complications. Therefore, it is crucial to investigate the pathogenesis of these cataracts. Advancements in molecular biology have made the effect of FRGs on cataract progression a key area of research. Wei et al. [14] explored the vulnerability of LECs to ferroptosis. Extremely low doses of Erastin and RSL3 triggered ferroptosis in HLECs (FHL124) and mouse LECs in vivo. Concurrently, the consumption of intracellular glutathione significantly sensitizes cells to ferroptosis, especially under the stimulation of RSL3. In addition, both HLECs and mouse lens epithelium show age-related susceptibility to ferroptosis. However, most previous studies have been limited to animal or cellular experiments. We used diagnostic biomarkers to identify pathological changes in patients with cataracts. Currently, validation of biomarkers associated with cataracts is the primary focus of research. In 2021, our team used bioinformatics methods to screen for biomarkers related to cataracts caused by Brg-1 mutations [34]. However, cataracts caused by Brg-1 mutations are clearly not the predominant types of cataracts encountered in clinical practice. More robust and comprehensive designs should be considered when using bioinformatics methods to investigate diseases.

In this study, we combined two cataract datasets, for the first time and subjected them to a comprehensive analysis with ferroptosis genes collected from a database to screen for the central genes related to ferroptosis in cataracts. By taking all |logFC| > 0.25 and p (adjust) < 0.05, DEGs and FRGs intersections, a total of 19 FRGs were obtained. We performed enrichment analysis on these FRGs, and the results showed that the above FRGs were primarily enriched in oxidative stress, glutathione metabolism, the ATF4 activated gene response to endoplasmic reticulum stress, and the MAPK pathway. Oxidative stress is crucial in aging-related processes in the body, such as the development of age-related cataracts (ARC). The formation of cataracts is promoted by both the generation of ROS and the decrease in native antioxidants. The lens is continuously exposed to oxidative stress from various sources, including radiation, which damages proteins, lipids, polysaccharides, and nucleic acids. However, as the body ages, the build-up of oxidized lens components and the decline in the efficiency of repair mechanisms contribute to the development of cataracts [34]. In addition, human cataract lenses showed decreased sulfonyl and glutathione levels. Activities of glutathione reductase, glutathione peroxidase, and glucose-6-phosphate dehydrogenase were significantly reduced, a process driven primarily by oxidative stress [35]. Ma et al. [36] demonstrated the involvement of the endoplasmic reticulum stress response, mediated by the *ATF4* activated gene, in cataract development. The unfolded protein response (UPR), a regulatory mechanism associated with PERK, has also been implicated in cataract development. Both *Nrf2* and *ATF4*, which are downstream effectors of PERK signaling, are activated to manage dual stress convergence. This interaction drives specific enzymatic and non-enzymatic antioxidant mechanisms to maintain redox homeostasis. Therapies that restore *Nrf2* or *ATF4* expression could potentially slow the aging of LECs and the onset of ARC. Numerous studies have explored the role of the MAPK pathway in cataract development, and recent in vitro studies have reported improvements in oxidative stress-related damage by inhibiting MAPK through the regulation of NLRP3 inflammasomes [37].

Using different algorithms, we identified nine genes, namely I*L6*,* PTGS2*,* ATF3*,* TNFAIP3*,* MDM2*,* SOCS1*,* HMOX1*,* TIGAR*, and *SNAI2*, as candidate hub genes for cataracts. Furthermore, the relationship between these hub genes and cataracts was validated using RT-PCR. We found that the levels of *TIGAR*,* IL6*,* ATF3*, and *TNFAIP3* were considerably higher in the cataract group than those in the control group. To further understand the causal relationship between these hub genes and cataracts, we performed an MR analysis. This is the first study to investigate the causal relationship between the levels of hub genes (exposure) and the risk of cataracts (outcome) through a two-sample MR analysis using GWAS data on hub genes and cataracts. *TIGAR* and *IL6* were found to be causally linked to cataracts risk. We specifically selected individuals from the European population in our dataset to eliminate any confounding factors between SNPs and *TIGAR*, *IL6*, and cataracts. In conclusion, we performed the MR-Egger regression test to confirm the stability of our results, which showed no evidence of directional horizontal pleiotropy.

*IL6* is a key participant in chronic systemic inflammation, and its expression levels change with age [38]. *IL6* is closely associated with cataracts. Dong et al. [39], by analyzing the serum of both patients with cataracts and normal individuals, revealed that the levels of *IL6*,* IL1β*,* CRP*, and *TNF1α* in the serum of patients with cataract were elevated compared to those in the control group. Additionally, the serum *IL6* expression level in older diabetic patients with cataracts was significantly higher than that in older non-diabetic patients with cataracts and healthy individuals [40]. The bioinformatics and RT-PCR results in the present study were consistent with those in these studies. In addition, our MR results further confirmed that *IL6* is a potential causal risk factor for cataracts.

*TIGAR* (TP53-induced glycolysis and apoptosis regulator) is induced by P53. This gene plays a crucial role in encoding regulatory enzymes for glycolysis and ROS detoxification. It can lower the fructose-2,6-bisphosphate level in cells, resulting in the suppression of glycolysis and an overall reduction in ROS levels within cells. These functions of *TIGAR* are related to its ability to protect cells from ROS-mediated apoptosis. *TIGAR* expression is closely associated with ferroptosis. Research has shown that *TIGAR* obstructs adenosine-triggered ferroptosis in human proximal tubular epithelial cells via the mTOR/S6KP70 signaling pathway [41]. In addition, in a study of intrahepatic cholangiocarcinoma, *TIGAR* knockdown reduced cell motility (cell proliferation/migration/invasion/colony formation ability) and increased ROS and lipid peroxidation, suggesting that *TIGAR* knockdown induces ferroptosis. This is related to the poor prognosis of patients with ICC and their resistance to ferroptosis [42]. Although there is currently no research related to *TIGAR* and cataracts, owing to the characteristics of cataract patients’ lenses, such as increased ROS formation, increased lipid peroxidation, and accumulation of redox-active iron in cells, it is probable that *TIGAR* plays a significant role in ROS production, lipid peroxidation, and ferroptosis in the lenses of cataract patients. The bioinformatics and RT-PCR results in this study showed that *TIGAR* significantly increased in the cataract group compared to that in the control group. MR resonance imaging revealed that *TIGAR* decreased the risk of cataracts. *TIGAR* is closely related to ferroptosis. It may play a protective role by blocking LEC ferroptosis through certain mechanisms, such as inhibiting glycolysis or reducing intracellular ROS levels. Compared with that in the control group, the observed increase in *TIGAR* in the local tissue of the disease group may represent a self-protection mechanism. Investigation of the specific mechanism of action is indeed a crucial aspect of further investigation.

However, our study has some limitations. One of the main limitations is the scarcity of microarray data in the field of cataracts, which restricts the use of only two datasets. The results would be more persuasive if additional cataract-related datasets were incorporated. Furthermore, our study was limited to the use of bioinformatic methods to examine the key genes associated with cataract development and their potential functions, with RT-PCR being the only method of verification. Future studies should include additional biological experiments to validate the specific mechanisms underlying the identified key genes.

## Conclusion

In summary, based on integrated bioinformatics analysis, in vitro experimental verification, and MR analysis, we determined two ferroptosis-specific expressed genes, *TIGAR* and *IL6*, as potential biomarkers for the diagnosis and treatment of cataracts. This may contribute to the development of targeted therapies for cataracts and offer further insights into the molecular mechanisms underlying cataract-risk genes.

## Electronic supplementary material

Below is the link to the electronic supplementary material.

Supplementary Material 1

## Data Availability

GSE153022 and GSE161701 are available at the Gene Expression Omnibus database (https://www.ncbi.nlm.nih.gov/geo/). The data of MR analysis can be found from IEU OpenGWAS (https://gwas.mrcieu.ac.uk/).

## Abbreviations

BP: Biological processes
CC: Cellular components
Closeness: Closeness Centrality
DEG: Differentially expressed genes
Degree: Degree Correlation
EPC: Edge Percolated Component
FRG: Ferroptosis-related gene
FRDEG: Ferroptosis-related differentially expressed gene
GEO: Gene Expression Omnibus
GO: Gene ontology
GSEA: Gene set enrichment analysis
HLEC: Human LECs
IV: Instrumental variables
IVW: Inverse variance weighting
KEGG: Kyoto Encyclopedia of Genes and Genomes
LEC: Lens epithelial cell
MR: Mendelian randomization
MCC: Maximal Clique Centrality
MF: Molecular functions
MNC: Maximum Neighborhood Component
PCA: Principal component analysis
PPI: Protein–protein interaction
ROS: Reactive oxygen species
SNP: Single nucleotide polymorphisms
