# Supplementary material for: From gene expression to causal associations: investigating the role of ferroptosis in cataract development
Source: BMC Med Genomics. 2025 Jul 1;18:111. doi: 10.1186/s12920-025-02177-6 (PMC12219257; doi:10.1186/s12920-025-02177-6)
Supplement: Supplementary file 1 — Supplementary Material 1 [file 12920_2025_2177_MOESM1_ESM.docx]

**Table S1 The name of 562 ferroptosis-related genes**

| ACSL4 AKR1C1 AKR1C2 AKR1C3 ALOX15 CBS CISD1 DPP4 GCLC GCLM GPX4 GSS HMGCR HSPB1 LPCAT3 NCOA4 SAT1 SLC7A11 FDFT1 TFRC TP53 AIFM2 PHKG2 FTH1 STEAP3 ACSL3 NOX1 SLC1A5 IREB2 HMOX1 ATP5MC3 CARS CS CRYAB RPL8 EMC2 HSBP1 ACACA ZEB1 ABCC1 G6PD PGD ACSF2 BECN1 YAP1 OTUB1 FBXW7 ATF4 PARK7 WWTR1 PRDX6 PRKAA1 MDM2 LCN2 SCD RELA PKM IL6 HMGB1 SIRT3 SP1 ELAVL1 TNFAIP3 PANX1 ATF3 KRAS CUL4B PROM2 NEDD4L EMP1 YTHDF2 SOCS1 HSPA5 HELLS ENO1 LAMP2 STAT3 DECR1 ECH1 DHODH STK11 PLA2G6 HNRNPD TIGAR PCBP3 FSCN1 NF2 XRCC6 CCT3 ATM TNF SKP2 SLC39A7 ATG16L1 ZFP36 H2AC1 MEF2C BAP1 TGFB1 ADCY10 SSBP1 ERBB2 CTNNB1 CTSB TLR4 YWHAE NR1H4 CDC25A RACK1 RPL7 RPS3A IDH1 PTEN GJA1 GSK3B PRDX1 CAV1 PRKCB SREBF1 FUS HAMP LRFN5 PRRX2 PSEN1 AHCY GGT1 SIRT1 SPARC GPD2 MAT2A MYCN USP14 UBE2G2 FOXC1 STYK1 UBE2J2 NKAP PIR RBMS1 SESN2 MIOX MARCHF6 VDAC1 IDH2 NFKB1 KAT5 PHGDH DLD KDM1A HNRNPA1 JUN LIFR STUB1 MDM4 SOX2 DKK1 LTBP2 SNAI2 BMAL1 GSTZ1 SLC6A14 GPX3 HCAR1 RETREG1 YTHDC2 USP35 MITD1 DDR2 YY1 SPHK1 BIRC5 EFEMP1 HNRNPA2B1 LONP1 WDR5 FBLN1 CD82 PDK4 RBMX ASAH2 HNRNPC MGST1 CUL9 ISCA2 USF2 HRNR SMG9 TRIM7 HDDC3 YY2 CISD3 ESR1 EP300 CREB1 KDM3B KDM5C TFAP2A DDB1 ERCC6 UBE2D3 ALAS2 CCL5 CUL4A NGLY1 RBX1 H1-4 NFE2L1 SETDB1 PDSS2 SEMA5A CYB5R1 GABARAPL1 H1-5 ABCB10 DCAF8 H1-2 CYGB H1-3 UFL1 COX7A1 H1-1 PAQR3 COMMD10 WDR76 H1-10 USP7 NEDD4 ABCB6 CX3CL1 FXN LTF PYCR1 ACADSB ADIPOR1 FMR1 KDM4A H2AX JAM3 SLC25A10 YME1L1 ETV4 PLIN2 RHOT1 CSRP2 SHARPIN STOML2 CIRBP DAZAP1 METTL14 BNC1 FBXO31 GABPB1 C19orf12 ZSCAN25 ALKBH5 SLIRP SCGB1D2 PEDS1 TMED8 C8orf76 SQSTM1 NOTCH2 TRPV4 TYRO3 ATF6 CA9 FPR2 FZD7 SIRT6 ABCC5 BSG FXR1 TTBK2 CEP290 IGF2BP3 ITGB8 LASP1 APOC1 COPZ1 HLF RNF182 PCDHB14 EGFR PDGFRB PIM1 IGF2BP2 DPEP1 FABP4 FABP1 GALNT14 ALG3 CDK14 DNAJB6 ME1 CHMP1A ARPC1A KLF2 QSOX1 SOX15 ITGA6 PRKAA2 MAPK1 MYC LDHA PCNA ACTB ALB EGLN1 HSPA8 KRT18 ANXA2 MAP3K5 MDH2 TPM1 HIF1A FH KRT14 PHB1 TPM3 ANXA5 ATP6V1E1 CTSZ LGALS3 GNB1 PRKCSH ANP32A CARS1 EEF1A1 KRT16 KRT19 RPS3 RPS6 PSMA1 ANXA4 EIF4H KRT6B PSME1 RPL7A RPS2 SNAP29 CLTB PSMD8 STOM TXNL1 MYL12A TPM4 FKBP3 TPD52L2 ANP32B DECR2 TRIM26 DNAJC9 PYM1 CCDC124 BEX1 CDH2 EPAS1 HPCAL1 RB1 G3BP1 HILPDA G0S2 BCAT2 GDPD5 MTOR LPIN1 PRC1 HSPA9 HADHA RUVBL1 CBX3 NAP1L1 TGFBR1 SNCA UCHL1 GSTP1 VIM PGK1 TUBA1A UBA1 ALDOA FASN GPI MYH9 TUBB4A YWHAG ANXA1 ENO2 RPL5 ACVR1B EIF4A1 GNB3 HSP90B1 LDHB RPSA SMC3 YWHAB YWHAZ ATP5F1A CFL1 DLST DPYSL2 HP MYH10 PRDX2 VAPB ACTC1 CCT5 CKB EWSR1 PDIA3 TRIM28 XRCC5 ATP5F1B DUT PROK2 PTBP1 RPA2 RPL10 RPL15 RPL26 BANF1 CNBP ITIH3 LRPPRC DHX9 PDIA4 RPL13A RPLP0 RPS13 RPS7 VAPA CCT4 CCT6A CCT8 HMGN1 HNRNPL HNRNPM KHDRBS1 MAP4 NOLC1 PLXNA4 RPL34 RPLP2 SNRPA SNU13 PDIA6 PFN2 RPL30 SNRPD2 YBX1 FAM120A NACA RPS4X SNRPD1 ALYREF HNRNPA3 MUC16 MYL6B RUFY1 SLC25A31 USP44 WBP11 WTAP CNTNAP4 ERH KSR2 NGB RPL38 SNRPC SNRPF TBC1D5 YTHDF3 RPL36A ABRAXAS2 ACTBL2 RUFY3 TEX264 IGKC H2AC21 KBTBD3 RTL1 SRSF8 IGHG4 KIAA1614 TUBA4B IGKV1-5 IGLV3-19 NET1 AIFM1 PDCD1 NDUFS7 LPIN2 SIAH2 CIAO1 LPIN3 CIAO2A OTUD1 SHH TGFB2 GFRA1 PKN2 UBR2 AURKA CDKN2A RIPK1 TRPV1 BRAF CASP8 SF3B2 MCU ALOX5 ALOX12 CD44 CHAC1 FANCD2 GLS2 MT1G PTGS2 ACO1 NFS1 PEBP1 SQLE FADS2 NFE2L2 KEAP1 NQO1 GOT1 SLC40A1 SLC3A2 VDAC3 POR PCBP1 ATG7 FTMT GCH1 MAP1LC3B CP MAP1LC3C PCBP2 NOX4 VDAC2 ATG5 MAP1LC3A ACSL1 TF BACH1 PRNP FTL TXNRD1 CTH CYBB SLC11A2 SLC39A14 SLC39A8 ACSL5 SLC38A1 ACSL6 CHMP5 COQ2 SAT2 CHMP6 |
| --- |

**Table S2 Primer information.**

| Target name | Primer | |
| --- | --- | --- |
| β-Actin | F | CATGTACGTTGCTATCCAGGC |
|  | R | CTCCTTAATGTCACGCACGAT |
| IL6 | F | ACTCACCTCTTCAGAACGAATTG |
|  | R | CCATCTTTGGAAGGTTCAGGTTG |
| PTGS2 | F | CTGGCGCTCAGCCATACAG |
|  | R | CGCACTTATACTGGTCAAATCCC |
| ATF3 | F | CCTCTGCGCTGGAATCAGTC |
|  | R | TTCTTTCTCGTCGCCTCTTTTT |
| TNFAIP3 | F | TCCTCAGGCTTTGTATTTGAGC |
|  | R | TGTGTATCGGTGCATGGTTTTA |
| MDM2 | F | GAATCATCGGACTCAGGTACATC |
|  | R | TCTGTCTCACTAATTGCTCTCCT |
| SOCS1 | F | CACGCACTTCCGCACATTC |
|  | R | TAAGGGCGAAAAAGCAGTTCC |
| HMOX1 | F | AAGACTGCGTTCCTGCTCAAC |
|  | R | AAAGCCCTACAGCAACTGTCG |
| TIGAR | F | ACTCAAGACTTCGGGAAAGGA |
|  | R | CACGCATTTTCACCTGGTCC |
| SNAI2 | F | CGAACTGGACACACATACAGTG |
|  | R | CTGAGGATCTCTGGTTGTGGT |

**Table S3 Results of GO and KEGG Enrichment Analysis**

| Ontology | ID | Description | Gene Ratio | Bg Ratio | p value | p. adjust |
| --- | --- | --- | --- | --- | --- | --- |
| BP | GO:0006979 | response to oxidative stress | 7/18 | 433/18800 | 8.37e-08 | 0.0001 |
| BP | GO:0062197 | cellular response to chemical stress | 6/18 | 332/18800 | 4.5e-07 | 0.0003 |
| BP | GO:2001233 | regulation of apoptotic signaling pathway | 6/18 | 370/18800 | 8.48e-07 | 0.0004 |
| BP | GO:0000302 | response to reactive oxygen species | 5/18 | 203/18800 | 1.07e-06 | 0.0004 |
| BP | GO:2001234 | negative regulation of apoptotic signaling pathway | 5/18 | 230/18800 | 1.97e-06 | 0.0005 |
| CC | GO:0031968 | organelle outer membrane | 3/19 | 232/19594 | 0.0014 | 0.0396 |
| CC | GO:0019867 | outer membrane | 3/19 | 234/19594 | 0.0014 | 0.0396 |
| CC | GO:0005901 | caveola | 2/19 | 82/19594 | 0.0028 | 0.0527 |
| CC | GO:0044853 | plasma membrane raft | 2/19 | 113/19594 | 0.0053 | 0.0740 |
| MF | GO:0043130 | ubiquitin binding | 2/19 | 96/18410 | 0.0043 | 0.0924 |
| MF | GO:0032182 | ubiquitin-like protein binding | 2/19 | 116/18410 | 0.0063 | 0.0924 |
| MF | GO:0002020 | protease binding | 2/19 | 136/18410 | 0.0085 | 0.0924 |
| MF | GO:0020037 | heme binding | 2/19 | 139/18410 | 0.0089 | 0.0924 |
| MF | GO:0046906 | tetrapyrrole binding | 2/19 | 149/18410 | 0.0102 | 0.0924 |
| KEGG | hsa00480 | Glutathione metabolism | 3/16 | 57/8164 | 0.0002 | 0.0186 |
| KEGG | hsa04657 | IL-17 signaling pathway | 3/16 | 94/8164 | 0.0007 | 0.0340 |
| KEGG | hsa04625 | C-type lectin receptor signaling pathway | 3/16 | 104/8164 | 0.0010 | 0.0340 |
| KEGG | hsa04668 | TNF signaling pathway | 3/16 | 112/8164 | 0.0012 | 0.0340 |
| KEGG | hsa05206 | MicroRNAs in cancer | 4/16 | 310/8164 | 0.0026 | 0.0518 |

GO, Gene Ontology; BP, Biological Process; CC, Cellular Component; MF, Molecular Function; KEGG, Kyoto Encyclopedia of Genes and Genomes.

**Table S4 Results of Combined Datasets GSEA**

| ID | Set Size | Enrichment Score | NES | p value | p. adjust | q values |
| --- | --- | --- | --- | --- | --- | --- |
| REACTOME_PYROPTOSIS | 22 | 0.69504536 | 2.04583899 | 0.0005987 | 0.00844122 | 0.00673194 |
| REACTOME_ATF4_ACTIVATES_GENES_IN_RESPONSE_TO_ENDOPLASMIC_RETICULUM_STRESS | 26 | 0.66818346 | 2.05237258 | 0.00062272 | 0.00860884 | 0.00686562 |
| WP_APOPTOSIS | 74 | 0.66439974 | 2.50994197 | 2.9346E-10 | 3.6752E-08 | 2.931E-08 |
| REACTOME_NEGATIVE_REGULATION_OF_MAPK_PATHWAY | 42 | 0.53821125 | 1.81493254 | 0.00448817 | 0.03747181 | 0.02988408 |
| WP_NOTCH_SIGNALING_PATHWAY | 51 | 0.55776707 | 1.9677855 | 0.00035883 | 0.00574392 | 0.00458082 |
| WP_WNT_SIGNALING_PATHWAY_AND_PLURIPOTENCY | 75 | 0.45903079 | 1.73372333 | 0.0039204 | 0.03406746 | 0.02716908 |
| WP_IL18_SIGNALING_PATHWAY | 191 | 0.55426619 | 2.36339663 | 1E-10 | 1.4193E-08 | 1.1319E-08 |
| WP_TP53_NETWORK | 17 | 0.71049533 | 1.95181861 | 0.00280704 | 0.02667943 | 0.02127706 |

GSEA, gene set enrichment analysis.

**Table S5. SNPs information of IL6 and TIGAR with cataract**

|  | **exposure** | | | | | **outcome** | |
| --- | --- | --- | --- | --- | --- | --- | --- |
| **SNP** | **effect allele** | **other allele** | **Beta** | **se** | **pval** | **se** | **pval** |
| **IL6** | | | | | | | |
| rs1038348 | A | G | -0.0761 | 0.0166 | 4.67E-06 | 0.1503 | 0.5606 |
| rs11264224 | C | A | 0.452 | 0.0123 | 1.00E-200 | 0.1292 | 0.0355402 |
| rs11729730 | A | G | -0.051 | 0.0106 | 1.37E-06 | 0.1238 | 0.6933 |
| rs1218552 | A | G | -0.0577 | 0.0103 | 2.21E-08 | 0.2183 | 0.678 |
| rs12415143 | A | G | -0.0514 | 0.0101 | 4.15E-07 | 0.1141 | 0.1061 |
| rs1467156 | A | C | 0.0749 | 0.0159 | 2.64E-06 | 0.1168 | 0.08051 |
| rs3775578 | C | T | 0.0711 | 0.0147 | 1.32E-06 | 0.126 | 0.2373 |
| rs61811421 | T | C | -0.2534 | 0.012 | 1.19E-98 | 0.1165 | 0.0443302 |
| rs7187473 | C | T | 0.0593 | 0.0121 | 9.08E-07 | 0.157 | 0.199 |
| rs72633650 | C | T | 0.3534 | 0.0128 | 1.17E-168 | 0.1462 | 0.3519 |
| rs78013587 | T | C | 0.2172 | 0.0469 | 3.67E-06 | 0.428 | 0.4709 |
| rs8002194 | C | T | 0.053 | 0.0116 | 4.70E-06 | 0.6188 | 0.6708 |
| rs9952221 | A | T | 0.0613 | 0.0129 | 1.94E-06 | 0.4179 | 0.2138 |
| **TIGAR** | | | | | | | |
| rs116844491 | A | G | 0.2871 | 0.0619 | 3.47E-06 | 0.1493 | 0.7381 |
| rs11732749 | C | G | -0.1155 | 0.0245 | 2.45E-06 | 0.117 | 0.922 |
| rs17690319 | A | G | 0.3254 | 0.0583 | 2.45E-08 | 0.2613 | 0.6026 |
| rs2964736 | T | G | 0.1268 | 0.0272 | 3.24E-06 | 0.2328 | 0.7515 |
| rs4930766 | C | T | -0.1784 | 0.0378 | 2.40E-06 | 0.1206 | 0.1791 |
| rs62143198 | A | G | 0.5725 | 0.0291 | 2.75E-86 | 0.1463 | 0.2824 |
| rs678950 | G | A | -0.1156 | 0.0249 | 3.47E-06 | 0.1226 | 0.2255 |
| rs76558876 | T | A | 0.1479 | 0.0322 | 4.47E-06 | 0.1522 | 0.1858 |
| rs9294052 | A | T | -0.1274 | 0.0272 | 2.95E-06 | 0.1125 | 0.6553 |

**Table S6. Heterogeneity and horizontal pleiotropy tests of IL6 and TIGAR on cataract**

| **Exposures** | **Q_1_ pval (IVW)** | **Q_2_ pval (MR-Egger)** | **I^2^** | **intercept** | **intercept pval** |
| --- | --- | --- | --- | --- | --- |
| IL6 | 0.860 | 0.985 | 0.0% | -0.147 | 0.225 |
| TIGAR | 0.505 | 0.443 | 0.0% | 0.032 | 0.615 |

Q_1_ pval: P-value of Q test from IVW method; Q_2_ pval: P-value of Q test from MR-Egger method.

Abbreviations: pval, P-value; Q, Cochran Q statistics; IVW, the inverse variance weighted method.
